# Assessing Calcium Intake in Postmenopausal Women

**Published:** 2009-09-15

**Authors:** Karen L. Plawecki, Ellen M. Evans, Mina C. Mojtahedi, Edward McAuley, Karen Chapman-Novakofski

**Affiliations:** Division of Nutritional Sciences, University of Illinois, Urbana-Champaign; University of Illinois, Urbana-Champaign, Urbana, Illinois; University of Illinois, Urbana-Champaign, Urbana, Illinois; University of Illinois, Urbana-Champaign, Urbana, Illinois; University of Illinois, Urbana-Champaign, Urbana, Illinois

## Abstract

**Introduction:**

Because foods fortified with calcium are increasingly available, the calcium content of calcium-fortified foods may not be adequately captured in traditional assessments of dietary intake, such as dietary records analyzed with commercially available software. The primary objective of our study was to design and test a calcium-focused food frequency questionnaire (CFFFQ) including foods naturally rich in calcium and calcium-fortified foods. Secondary objectives were to review calcium sources and adequacy of intake in black and in white postmenopausal women.

**Methods:**

We studied a convenience sample of 46 black and 139 white postmenopausal women (mean [SD] age 69.4 [5.8] years). Subjects completed a multiple-pass interview for 24-hour recall of foods eaten and the 46-item CFFFQ.

**Results:**

The correlation between measures for total daily calcium intake was moderately strong (r = 0.53, *P* < .001). The CFFFQ estimated greater total daily calcium intake than did the 24-hour recall (mean [SD], 1,021 [624] mg/d vs 800 [433] mg/d, *P* < .001). As daily calcium intake increased, the 24-hour recall increasingly underreported calcium (r = 0.41, *P* < .001) compared with the CFFFQ. Cross-tabulation and Χ^2^ analyses found that the CFFFQ had greater specificity for lower calcium intakes. For calcium classified by food groups, there was moderate correlation for dairy (r = 0.56, *P* < .001) and fruits (r = 0.43, *P* < .001). The CFFFQ overestimated mean total calcium compared with the 24-hour recall by 221 mg/d (*P* < .001), including within racial groups (195 mg/d for black women, *P* = .04, and 229 mg/d for white women, *P* < .001). Dairy was the primary calcium source for both groups (55% of intake for black women and 57% of intake for white women).

**Conclusion:**

The CFFFQ can be used to identify postmenopausal women with inadequate calcium intakes (<800 mg/d) and to identify key sources of dietary calcium. Older black women consume less daily calcium than do older white women.

## Introduction

As the prevalence of osteoporosis continues to increase, interventions targeting modifiable risk factors receive more emphasis from researchers, clinicians, and public health professionals. Although many nutrients are important to bone health, interventions largely focus on calcium intake (1). Clinicians often prescribe a calcium supplement but pay limited attention to dietary sources of calcium intake (2,3). Other interventions may target only dairy calcium (4,5). However, calcium fortification of foods broadens the possible sources of calcium so that adequate calcium intake may be achieved through a diverse diet.

Sources of dietary calcium may differ across cultures and ethnicities according to food preferences and tolerances. For example, blacks and Asians have a higher prevalence of lactose intolerance, which may lead to reduced dairy intake, but also have cultural food preferences, including not drinking milk at meals, that affect calcium intake (6). White women eat more cheese and drink more milk than do black or American Indian women (7). However, research specifically targeting calcium intake and food source is scarce. Previous research from our group investigating the role of socioeconomic status on calcium intake determined that black women consumed fortified grain products more frequently than did white women (8). Because fortified foods are increasingly available (9), the calcium content of calcium-fortified foods may not be adequately captured in traditional assessments of dietary intake, such as dietary records analyzed with commercially available software.

The primary aim of this study was to develop and evaluate the feasibility of a calcium-focused food frequency questionnaire (CFFFQ) that incorporates both natural and fortified sources of calcium. Because determining calcium intake has become more difficult as food fortification has become more common, this study compared 2 methods: an interview for 24-hour recall of calcium intake and the CFFFQ. Although supplements can be an important source of calcium, this project focused on food-derived calcium. Secondary aims were to compare food source of calcium between black and white women aged 60 years or older and evaluate the adequacy of calcium intake as assessed using the 24-hour recall and the CFFFQ. Postmenopausal women were studied because they are at high risk for osteoporotic fractures (1).

## Methods

### Participants

In 2005, we recruited a convenience sample of postmenopausal women aged 60 to 80 years who were enrolled in a parent study in the Urbana-Champaign area of Illinois (10) (assessing body composition, bone health, physical activity, physical function, and self-efficacy) to complete additional nutritional assessments. Exclusion criteria for the parent study were neurologic illness, orthopedic limitations, or cognitive limitations that precluded completion of all study testing procedures. All those in the parent study were invited to participate in the current study of evaluation of calcium intake. The human subjects institutional review board of the University of Illinois approved the research protocol, and all participants completed an informed consent form before data collection began.

### Calcium intake

The CFFFQ and a self-reported 24-hour recall were completed on the same day to assess calcium intake and food source. The CFFFQ is a 46-item food frequency survey focusing on calcium-rich foods. Foods on the questionnaire were chosen based on calcium content and those found in previous studies to be commonly consumed. Foods on the CFFFQ were categorized and presented according to representative food groups: dairy (6 foods), foods with dairy (5 foods), fruits (6 foods), vegetables (3 foods), grains (10 foods), meats (8 foods), and other foods (8 foods) (Appendix). Inclusion of fortified foods was based on availability for purchase in area grocery stores (both local and chain stores). Fortified foods were noted as such on the CFFFQ. A standard portion size was included for each specific food item. Participants entered quantity of food relative to daily, weekly, monthly, or yearly consumption. "Not eaten" was given as an option. We entered foods and servings into a database (Microsoft Access 2003, Redmond, Washington) that calculated calcium content on a daily basis. Double entry was used for quality assurance.

Participants met with researchers to complete the 24-hour recall. The researchers used a multiple-pass interview style to elicit complete information (11). Foods from the 24-hour recall were categorized into the same food groups as on the CFFFQ and were then entered in Nutritionist Pro version 2.3 (First DataBank, San Bruno, California) to obtain calcium values per day.

### Statistical analysis

We analyzed the data by using SPSS version 14.0 (SPSS, Inc, Chicago, Illinois). On the basis of the findings of the Kolmogorov-Smirnov test, the primary variables of interest were not normally distributed. Nonparametric tests were subsequently used to evaluate group or intake assessment differences (Spearman rank correlation and Mann-Whitney). We assessed the adequacy of calcium intake by comparing these 2 assessment methods to two-thirds of the Institute of Medicine's (12) adequate intake amount (used to determine inadequate intake) for women aged 51 years or older. Comparison of the adequacy classification as a measure of specificity of each calcium intake method was completed by using cross-tabulation to demonstrate the relationship between the 2 variables. Chi-square analysis was performed on the cross-tabulated variables. Significance was defined as α = .05.

## Results

Of 245 eligible women from the parent study, 185 chose to participate in our study. Of the 185, 46 were black (mean [SD] age, 68.0 [4.85] years) and 139 were white (70.0 [6.0] years).

### Comparison of CFFFQ to 24-hour recall

Calcium from the CFFFQ was significantly correlated with 24-hour recall for all food groups (*P < *.001) except for vegetables (*P =* .08) (Table 1). Estimates obtained with the 24-hour recall method were significantly lower than CFFFQ estimates of total daily calcium, dairy, foods with dairy products, fruits, and vegetables (Table 2). The CFFFQ estimate of mean total calcium compared with the 24-hour recall was higher by 221 mg/d (*P* < .001), including within racial groups (195 mg/d for black women, *P* = .04, and 229 mg/d for white women, *P* < .001).

There was no significant difference between the CFFFQ and 24-hour recall methods for calculated calcium intake for grains, meats, and "other" foods. This pattern was consistent for the total group and for black and white women separately (Table 2). The Bland-Altman plot illustrates the lack of agreement between methods of assessment for total daily intake (Figure). The positive correlation between the lack of agreement (error score on the Y-axis) and the daily intake indicates that for increasing levels of daily calcium intake, the 24-hour recall of calcium intake increasingly underestimated calcium intake compared with the CFFFQ (*r* = 0.41, *P* < .001).

**Figure.**
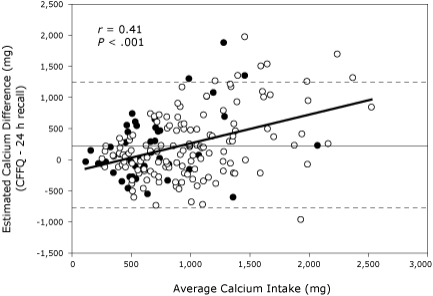
Scatterplot of agreement between the calcium-focused food frequency questionnaire (CFFFQ) and an interview for 24-hour recall of calcium intake. Estimated calcium difference is the difference in calcium intake found between the CFFFQ and the 24-hour recall. Average calcium intake is the average of calcium intake between the 2 methods. Open circles represent white women (n = 139) and closed circles represent black women (n = 46). The parallel solid and the broken lines represent the mean difference (2 SD) for all participants (221 [528] mg).

### Calcium adequacy assessment

The prevalence of inadequate intake (<800 mg) was 56% (n = 103) using the 24-hour recall and 45% (n = 83) using the CFFFQ method. Examining the cases where intake would be inadequate as measured by both tools, 64 (35%) women would be classified as having inadequate calcium intake. However, 39 (21%) women whose intake would be classified as adequate by the CFFFQ would be classified as inadequate by the 24-hour recall method. Only 19 (10%) who would be classified as having inadequate intake by the CFFFQ would have adequate intake as classified by the 24-hour recall. Compared with the self-reported 24-hour recall, the CFFFQ indicates more specificity for lower intakes.

### Comparison of intake and source between black and white women

Regardless of dietary assessment method used, white women had higher calcium intakes than black women. When using the CFFFQ, white women reported consuming approximately 43% more calcium than did black women (mean [SD] 1,104 [632] mg for white women vs 768 [531] mg for black women, *P* < .001). When using the 24-hour recall method, mean calcium intake for white women was approximately 52% higher than intake for black women (875 [429] mg vs 573 [365] mg, *P* < .001).

From the CFFFQ, the primary calcium source was dairy products (55% for black women and 57% for white women). White women obtained more calcium (630 [423] mg) from dairy than did black women (424 [373] mg, *P* < .004). Grains were the second highest calcium source, although grains provided a much lower percentage than dairy (13% of total calcium for each racial group). Calcium from grains primarily came from fortified foods. Dairy was also the primary calcium source when the 24-hour recall data were analyzed, and a significant difference in mean (SD) dairy calcium intake between racial groups was found (243 [273] mg for black women, 444 [360] mg for white women, *P* < .001).

## Discussion

Calcium intake has received increased attention in the last decade because of its role in bone health and as a modifiable risk factor for osteoporosis. The number of calcium-fortified food products being developed and marketed has increased substantially (9). Although labeling for "excellent" (>200 mg/serving) and "good" (100-200 mg/serving) sources of calcium are regulated by the Food and Drug Administration, there is no federal regulation regarding which foods can be fortified with calcium or the degree of fortification (13). Together, these factors make discerning calcium intake difficult for researchers, clinicians, dietitians, and consumers. The primary findings of this study are that the CFFFQ identifies low calcium intakes and identifies key sources of calcium, including calcium-fortified foods.

Other studies have included either no calcium-fortified foods (14-18), calcium-fortified mineral water (19), calcium-fortified juice alone (20), calcium-fortified juice and a grain product (21), or did not provide details regarding the inclusion of calcium-fortified foods in the assessment tool (5,7,22-26). In contrast, the CFFFQ is unique in providing 14 fortified food choices, including 1 in fruits, 8 in grains, and 5 in "other" food categories. Fortification of foods can contribute greatly to total calcium intake. From our data, inclusion of calcium-fortified foods in the diet has the potential to increase total daily calcium intake by 1,000 mg or more. Because calcium fortification of food products is not federally regulated, food items being fortified and levels of fortification vary at the discretion of the manufacturer. The lack of regulation may hinder the role that these foods can play in dietary calcium intake by affecting the consistency of the calcium content of the product.

The second unique feature of this study is the categorization of calcium intake estimates into food groups. Although total calcium intake is the primary focus in nutritional assessment studies, the effectiveness of nutrition education and osteoporosis interventions can be enhanced by focusing on calcium intake from the food groups the participants usually get their calcium. This information can be used to design more realistic and targeted nutrition education messages. In our study, the largest percentage of total calcium for both white and black women came from dairy, followed by grains and fruits. Although the grain category contained several fortified food items, the fruit category contained only 1 fortified food item.

Even though consumption of fortified foods was assessed, dairy was still the primary calcium source found in this study. An analysis (15) of food intake from the US Department of Agriculture's 1994-1996, 1998 Continuing Survey of Food Intakes by Individuals (CSFII) found that dairy contributed 42% of total calcium intake. A higher percentage of dairy contribution would have been expected from that study because it included no competing calcium-fortified foods. In addition, the CSFII reported 21% of total calcium from calcium-rich mixed foods (ie, foods with 2 or more items with calcium such as sandwiches or pizza). In our study, this category contributed only 4% to 5%. Ward et al (21) also reported that most calcium intake was derived from dairy, but a comparable calcium-rich mixed foods group is not identified. The food frequency assessment tool used by Ward et al (21) also ranked the fruits and vegetables group and grains as contributing 11% to 15% of total calcium, whereas the diet records used in the study estimated this contribution at 6% to 13%. Cook et al (15) included no calcium-fortified foods, and the Ward et al (21) study was limited to calcium-fortified juice and 2 grain products.

There is a scarcity of information in the literature regarding calcium intake for black and white women. In our study, mean total calcium intake among black women did not meet the adequate intake value whereas it did among white women. Other studies conclude that calcium intake is greater in white women than black women (27,28). However, an article published from the parent study of our study using the same CFFFQ found no difference in total, daily, dietary, or supplemental calcium (8). In that analysis, black and white women were matched on age, socioeconomic status, and education level (n = 33 per group). The findings suggest that racial differences in calcium intake are somewhat influenced by socioeconomic status and education level. Similarly, a comparison between black, white, and American Indian women also reported no significant difference in dietary calcium between black and white women (7).

In our study, grains were the second-highest calcium source for both black and white women. Grains are predominately calcium-fortified sources. This is consistent with findings from our previous work, which determined that dairy was the greatest source of calcium but more for white women, whereas black women consumed greater amounts of calcium-fortified grains (8). The prominent role of calcium-fortified foods in these studies reinforces the need for these sources to be carefully evaluated when measuring calcium intake.

### Limitations

The values from the CFFFQ were typically higher than those from the 24-hour recall, with the exception of vegetables and "other" foods. This finding may be due in part to the limitations of using a 24-hour recall. Women may have identified foods in the CFFFQ but did not happen to consume those foods on the specific day of the recall, except for vegetables. However, Chee et al (24) found a similar trend in comparing a calcium-rich food frequency questionnaire with a 3-day diary for postmenopausal Malaysian women.

Assessing dietary intake by any method has inherent limitations. Validating food frequency questionnaires can be complicated without biomarkers for comparison. Because most nutrients do not yet have reliable biomarkers, 24-hour recalls or food records are the usual standard instruments. Correlations between these methods are considered adequate within the range of 0.4-0.7 (29). Although most of the correlations between total calcium and calcium from food groups when comparing CFFFQ to 24-hour recalls are significant, only total calcium intake, dairy calcium intake, and fruits fall within this acceptable statistical range.

Differences in reported calcium intake when comparing the CFFFQ and 24-hour recall could be attributed to a lack of consumer awareness of calcium fortification when responding to the 24-hour recall and a positive respondent bias on the CFFFQ. For example, participants could have responded more positively concerning calcium-fortified foods if consumption of that food was seen as a positive health behavior. Because calcium-fortified foods can essentially double calcium intake, consumer awareness of their own calcium-fortified food product consumption and interviewer probe for each of these food items are essential for an accurate estimate of calcium intake. To assist both the researcher and clinician, software for dietary assessment needs to be updated to include the calcium-fortified foods.

Several food frequency questionnaires that assess calcium intake have shown strong correlation with food actually consumed (19,22-25,30,31). However, many studies report correlations but no difference between means or report findings regarding only total calcium intake and not calcium intake by individual food groups. In addition, many rapid assessment tools include either no or limited sources of calcium-fortified foods, and these foods may or may not be probed for on 24-hour recalls.

### Conclusions

Our results suggest that the CFFFQ could be used to determine inadequate intakes of calcium. Primary calcium sources for all women were dairy followed by grains, and calcium intake was higher in white women than in black women. The CFFFQ can be used to more accurately identify calcium intakes and usual calcium source, which is of interest because of the increasing availability of calcium-fortified foods. Calcium-fortified sources can go unreported in 24-hour recalls and diaries, leading to an underestimation of calcium intake. When 24-hour recalls are used to assess and quantify calcium intake, researchers and clinicians need to clarify with clients whether the foods they ate were fortified. The CFFFQ may be used to better quantify calcium intake, including both natural and fortified sources. Accurate assessment of calcium is critical in evaluating bone health risks. This information can aid in the development of effective interventions to increase calcium intake.

## Figures and Tables

**Table 1:** Correlation of Calcium-Focused Food Frequency Questionnaire to Interview for 24-Hour Recall of Calcium Intake for Black and White Women, 2005

Food Group	All Women (n = 185)		Black Women (n = 46)		White Women (n = 139)	
	r^a^	*P* value	r^a^	*P* value	r^a^	*P* value
Dairy	0.56	<.001	0.42	.004	0.56	<.001
Foods with dairy	0.18	.015	0.03	.83	0.20	.02
Fruits	0.43	<.001	0.32	.03	0.48	<.001
Vegetables	0.13	.078	0.10	.52	0.14	.09
Grains	0.25	.001	0.25	.10	0.27	.002
Meats	0.18	.013	0.05	.76	0.22	.008
Other foods	0.27	<.001	0.04	.77	0.28	.001
**Total calcium**	0.53	<.001	0.29	.05	0.53	<.001

a Spearman rank correlation.

**Table 2:** Difference Between Calcium-Focused Food Frequency Questionnaire (CFFFQ) and Interview for 24-Hour Recall of Calcium Intake for All Women (n = 185), Black Women (n = 46), and White Women (n = 139), 2005

**Food Group**	CFFFQ Mean (Interquartile Range), mg	24-Hour Recall Mean (Interquartile Range), mg	*P* value^a^
**Dairy**			
All women	579 (257-812)	394 (108-580)	<.001
Black women	424 (184-519)	243 (7-365)	<.001
White women	630 (184-519)	444 (153-659)	<.001
**Foods with dairy products**			
All women	40 (16-55)	40 (0-0)^b^	<.001
Black women	33 (5-44)	29 (0-0)^b^	.001
White women	43 (20-60)	43 (0-0)^b^	<.001
**Fruits**			
All women	99 (8-159)	57 (6-58)	.002
Black women	99 (3-165)	44 (4-44)	.03
White women	99 (8-156)	62 (7-62)	.03
**Vegetables**			
All women	24 (6-30)	64 (19-95)	<.001
Black women	24 (5-30)	47 (6-58)	.01
White women	24 (6-30)	71 (24-100)	<.001
**Grains**			
All women	132 (17-153)	103 (35-119)	.73
Black women	103 (18-125)	85 (26-116)	.99
White women	142 (16-167)	109 (37-122)	.72
**Meats**			
All women	93 (14-121)	82 (29-100)	.98
Black women	63 (5-74)	83 (24-109)	.11
White women	103 (18-127)	82 (31-100)	.39
**Other foods**			
All women	54 (7-75)	59 (10-65)	.41
Black women	23 (7-75)	44 (5-38)	.29
White women	64 (11-77)	64 (13-71)	.75
**Total calcium**			
All women	1,021 (542-1,323)	800 (460-1,057)	<.001
Black women	768 (381-956)	573 (311-695)	.04
White women	1,104 (592-1,449)	875 (551-1,112)	<.001

a Mann-Whitney.

a The dispersion of data reflects value concentration at high and low values rather than within the 25th-75th quartile range.

## Appendix: Calcium-Focused Food Frequency Questionnaire

Instructions: We would like to know how often you eat foods that are high in calcium. For each of these questions choose the appropriate time frame, and fill in the frequency.

For instance, if the question was — How often do you eat cake? — and you eat cake once per month, under "Per month" you would write "1" and leave the other columns blank.

**Table:**

**Food Group**	Per Day	Per Week	Per Month	Per Year	Don't Eat
cake			1		

If the question was — How often do you eat bread? — and you eat 4 slices of bread per day, under "Per day" you would write 4, and leave the other columns blank.

**Table:**

**Food Group**	Per Day	Per Week	Per Month	Per Year	Don't Eat
Bread, slice	4				

Some of these questions may be hard to "guess at." Just give your best estimate.

Quick key to portion sizes:

1/2 cup = tennis ball

1 cup = closed fist

1 oz = 1 piece of string cheese

**Table:**

**Food Group**	Per Day	Per Week	Per Month	Per Year	Don't Eat
**Dairy**					
Milk, 1 cup (including milk added to cereal, shakes, coffee, etc)					
Yogurt, 1 cup					
Aged cheese, 1 oz (eg, cheddar, Swiss, provolone, Monterey Jack, Colby)					
Cheese food, 1 oz (American, Velveeta)					
Cottage cheese, 1/2 cup					
Ice cream, frozen yogurt, 1/2 cup					
**Other foods using dairy products**					
Pizza, 1/8 of 15-inch pizza					
Lasagna, 1 cup or 8 oz					
Enchiladas, tacos, 1					
Macaroni and cheese, 1 cup					
Soups made with milk, 1 cup					
**Fruits**					
Orange juice with calcium^a^, 1 cup					
Orange, whole					
Papaya, 1 cup					
Rhubarb, 1 cup					
Raisins, 1 cup					
Pear halves, dried, 10 each					
**Vegetables, cooked**					
Broccoli, 1/2 cup					
Bok choy, 1/2 cup					
Greens (collard, turnip, mustard, spinach), 1/2 cup					
**Grains**					
Total cereal, 1 cup					
Special K Plus, 1 cup					
Other fortified^a^ cereal (eg, Basic 4, Life Cinnamon), 1 cup					
Cereal, 1 cup					
Fortified^a^ instant oatmeal, 1 cup					
Fortified^a^ graham crackers (eg, Teddy Grahams), 24 pieces					
Fortified^a^ cereal bars, 1 bar					
Fortified^a^ bread, 1 slice					
Corn bread, 1					
Waffles (homemade, from mix, frozen^a^), 1					
**Meats and meat alternatives**					
Sardines, 3.5 oz					
Oysters, clams, 20					
Crab legs, 1 cup					
Anchovies, 10					
Legumes/beans, 1/2 cup					
Almonds, 1/4 cup					
Mixed nuts, 1/4 cup					
Tofu set with calcium, 1/2 cup					
**Others**					
Fortified^a^ Crystal Light, 1 cup					
Fortified^a^ Country Crock margarine, 1 tbsp					
Cheesecake, 1/8 of cake					
Cream pies (including pumpkin), 1/8 of pie					
Chocolate bar (1.5 to 2.15 oz)					
Slim-Fast, can^a^, 325 mL					
Slim-Fast, powder mixed with water^a^, 1 cup					
Slim-Fast, powder mixed with milk^a^, 1 cup					

a Fortified with added calcium.

Nutrition Interview (voluntary): If willing, please answer the following:

Name:

Phone number:

Email address:
